# Super Subtotal Gastrectomy: A Novel Reconstruction Concept for Upper Gastric Cancer That Preserves the Fornix

**DOI:** 10.1002/ags3.70030

**Published:** 2025-05-08

**Authors:** Kohei Fujita, Hiroyuki Sagawa, Sunao Ito, Ryo Ogawa, Shuji Takiguchi

**Affiliations:** ^1^ Department of Gastroenterological Surgery Nagoya City University Graduate School of Medical Sciences and Medical School Nagoya Aichi Japan

**Keywords:** esophagogastric junction, gastrectomy, stomach neoplasms

## Abstract

The incidence of upper‐third gastric cancer is rising, necessitating proximal gastrectomy or total gastrectomy in most patients. However, surgical removal of the fornix, a major site for ghrelin secretion, often results in reduced appetite and weight loss post‐surgery. To address this issue, we devised a resection approach aimed at preserving ghrelin secretory sites. Here, we introduce a novel technique for treating upper‐third gastric cancer near the esophagogastric junction: super subtotal gastrectomy (SSTG). During distal gastrectomy assisted by robotics, lymph node dissection was performed. Endoscopic confirmation of the tumor site guided the design of the gastrectomy line. Using a linear stapler, the stomach was dissected from the greater curvature fold to the angle of His. The specimen was then extracted through a precise incision from the angle of His to the right side of the esophagus, partially resecting the esophagogastric junction. Suturing of the open lumens of the esophagus and stomach was performed to repair the remaining stomach. Closure of the diaphragmatic crus prevented esophageal hiatal hernia. Reconstruction was achieved through Roux‐en‐Y reconstruction. SSTG offers the advantage of maintaining an oral margin beyond the esophageal junction while preserving the fornix. In the SSTG group, the median operative time was 333 min (range: 257–354), with a blood loss of 79.5 mL (range: 20–141). No serious intraoperative complications were observed. Our proposed SSTG technique enables the preservation of the fornix even in cases of upper‐third gastric cancer located closer to the esophagogastric junction than was previously possible.

## Graphical abstract

We developed a novel super subtotal gastrectomy (SSTG) technique for upper‐third gastric cancer near the esophagogastric junction. This method preserves the gastric fornix, a major site of ghrelin secretion, while maintaining an oral margin beyond the esophageal junction. SSTG offers a potential solution for reducing postoperative complications such as weight loss and appetite reduction.

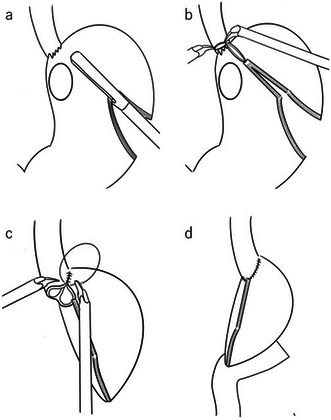

## 1 Introduction

The incidence of upper‐third gastric cancer is rising, necessitating diverse surgical approaches. Surgeons and facilities choose from options like proximal gastrectomy, total gastrectomy, and subtotal gastrectomy. Subtotal gastrectomy preserves the fornix [1], crucial for ghrelin secretion, which promotes better postoperative nutritional status and fewer complications, including reflux esophagitis, compared with total and proximal gastrectomies [1, 2, 3, 4]. However, when tumors near the esophagogastric junction require extensive excision, proximal or total gastrectomies become necessary.

Ghrelin‐producing cells have been observed in the fundic gland, mainly in the upper body and greater curvature [1]. This region secretes ghrelin, an appetite‐stimulating hormone [5], suggesting that preserving it might mitigate postoperative weight loss.

Robot‐assisted gastrectomy (RAG) is increasingly popular for the minimally invasive treatment of gastric cancer [6], enabling suturing techniques that are challenging to use with laparoscopic methods and broadening surgical options.

In this study, we introduce a novel surgical approach for upper‐third gastric cancer proximal to the esophagogastric junction, involving partial resection of the esophagogastric junction while preserving the fornix. This technique facilitates tumor excision while conserving the fornix region. We present the technical details and initial clinical outcomes of this fornix‐sparing resection, the first reported for upper‐third gastric cancers adjacent to the esophagogastric junction.

## 2 Surgical Technique

### 2.1 Patients

Between June 2023 and December 2023, four patients underwent super‐subtotal gastrectomy (SSTG) at the Department of Gastroenterological Surgery at Nagoya City University, Aichi, Japan. The study was approved by the Institutional Review Board of the Graduate School of Medical Sciences, Nagoya City University (approval number: 60‐18‐0008). The indications for SSTG were: (1) the tumor had not invaded the esophagogastric junction; (2) the tumor was centered on the lesser curvature; and (3) no metastasis occurred to the lymph nodes of the fornix.

The median age of the patients was 69.5 years (range: 54–72 years); all patients were male. This procedure is indicated in patients with gastric cancer localized to the lesser curvature of the upper‐third of the stomach. The procedure was performed after informed consent was obtained from all patients.

### 2.2 Technique

**FIGURE 1:**
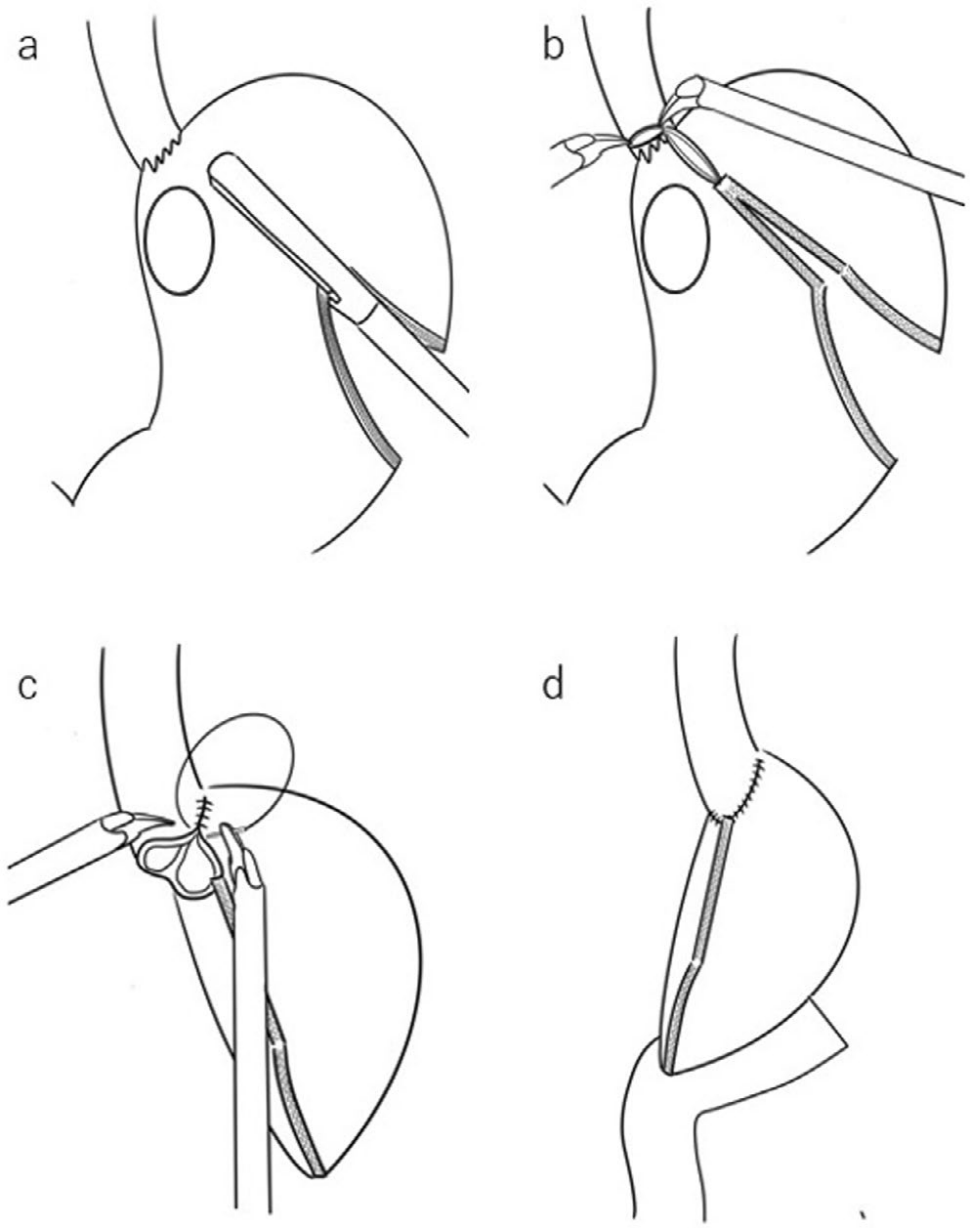
Schemas of super‐subtotal gastrectomy. (a) Stapling the stomach from the greater fold to the angle of His. (b) Resection of the right wall of the esophagogastric junction, while leaving the left wall intact. (c) Resection of the stomach containing the tumor, followed by suturing the esophagus and stomach closed. (d) Performance of Roux‐en‐Y reconstruction.

Under general anesthesia, the patients were positioned with the right leg in an open‐leg position to prevent arm interference. First, a 12‐mm port was inserted into the umbilicus using an open technique. All procedures were performed using the da Vinci Xi robotic platform (Intuitive Surgical, Sunnyvale, CA) or the Hinotori Surgical Robot System (Medicaroid Corporation, Kobe, Japan). D1+ or D2 dissection during distal gastrectomy (Japanese Gastric Cancer Treatment Guidelines, 14th Edition) was performed with robotic assistance. Herein, we present a schematic diagram of the SSTG procedure (Figure 1). The tumor site was confirmed endoscopically; a gastrectomy line was designed along the tumor location.

**FIGURE 2:**
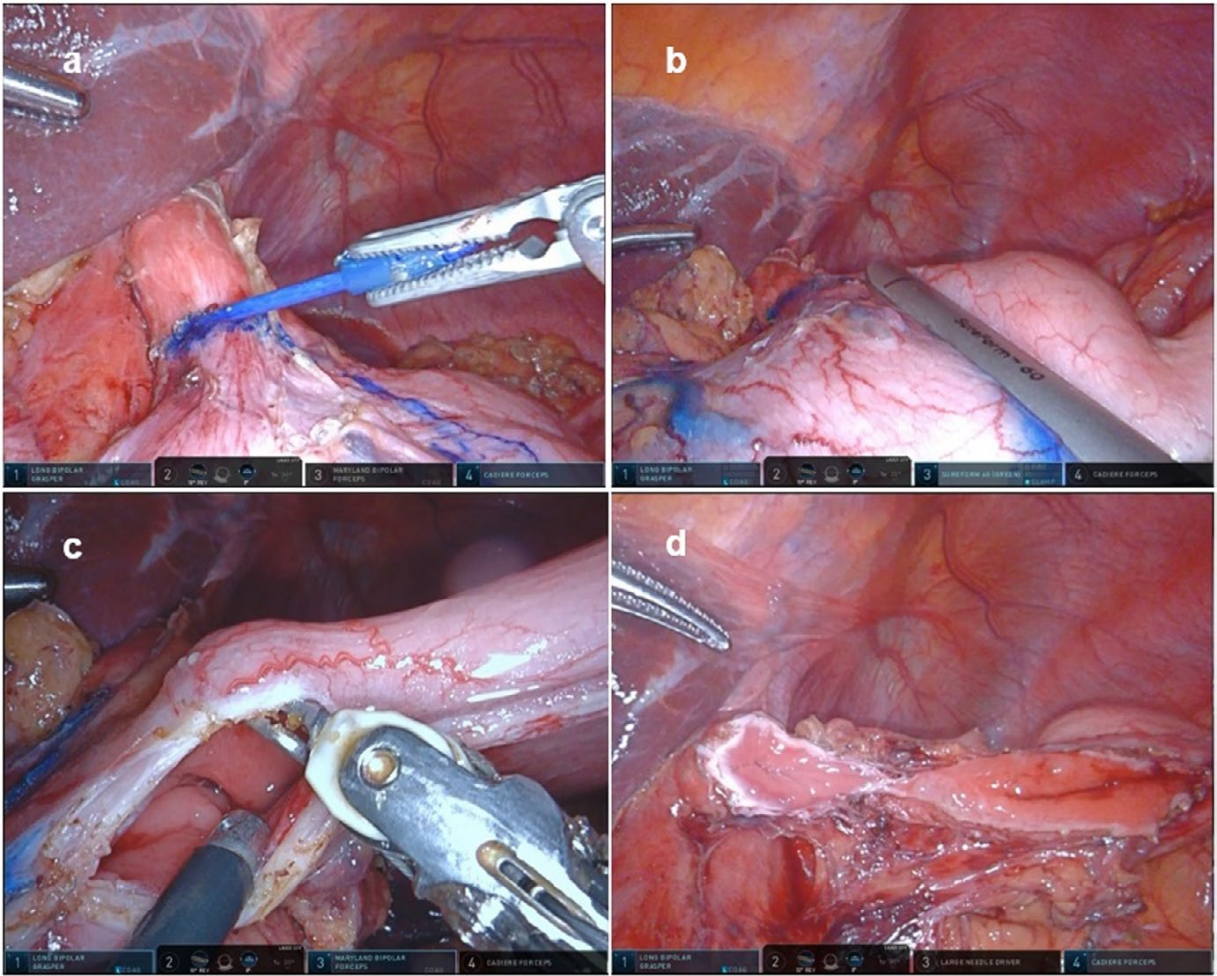
Partial resection of the esophagogastric junction. (a) Marking the line for the partial resection of the right wall of the esophagogastric junction. (b) Separating the stomach with an automatic suturing device along the marked line. (c) Resection of the stomach along the planned resection line while checking the lumen. (d) View after resection.

The resection line was designed from the right side of the esophagogastric junction toward the greater curvature to ensure an oral margin for the tumor located on the lesser curvature (Figure 2a). The stomach was dissected with a linear staple from the planned line of dissection of the greater fold to the angle of His (Figure 2b). The specimen was removed through a sharp incision from the angle of His to the right side of the esophagus, partially resecting the esophagogastric junction (Figure 2c). The remaining resection edges of the esophagus and stomach formed a shape resembling that of eyeglasses (Figure 2d). During the procedure, suction of the stomach contents was required to prevent spillage.

**FIGURE 3:**
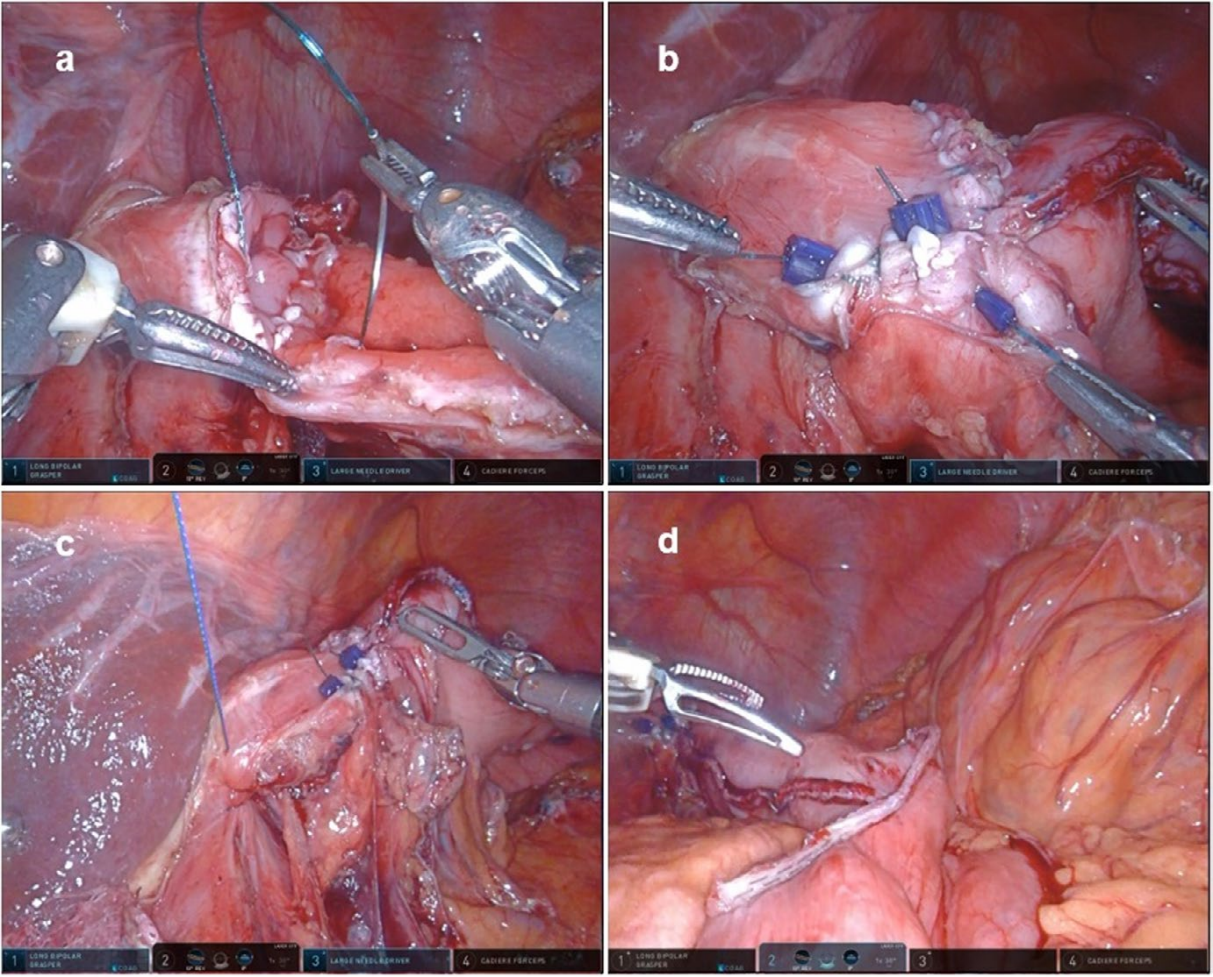
The reconstruction process following resection. (a) Suture closure of the esophagus and stomach from the posterior wall. (b) View after the sutures are completed. (c) Suturing and closure of the crus of the diaphragm. (d) View after anastomosis of the residual stomach and jejunum.

The open lumens of the esophagus and stomach were sutured with an absorbable barbed suture; the remaining stomach was repaired (Figure 3a,b). As a precaution during the procedure, while suturing the esophagus and stomach, the anatomical length of the posterior wall of the stomach was typically longer than that of the esophagus. Based on experience, using a 2:1 ratio where the stomach is bitten more than the esophagus helped match the lengths and achieve a clean suture closure.

The crus of the diaphragm was then closed with a barbed suture to prevent esophageal hiatal hernia (Figure 3c), and reconstruction was performed with a Roux‐en‐Y reconstruction (Figure 3d); SSTG has the advantage of providing an oral margin beyond the esophageal junction and preserving the fornix (Video S1).

## 3 Results

To date, SSTG has been performed in four patients with small‐curvature gastric cancer in the upper third of the stomach, including one case of remnant gastric cancer post‐distal gastrectomy (Table 1). No significant intraoperative complications were reported. The median operative time for SSTG was 333 min (range: 257–354 min), with an estimated blood loss of 79.5 mL (range: 20–141 mL). Although all tumors were located near the esophagogastric junction, negative proximal margins were achieved. Postoperatively, all patients experienced prompt recovery, with clearance to consume clear liquids by postoperative day 2 and solid food by postoperative day 4. Notably, no major complications, such as anastomotic leakage, stenosis, or pancreatitis, occurred; there were no instances of mortality among the patients.

**TABLE 1:** Patient characteristics and surgical outcomes.

Case no.	Age (y)	Sex	Main lesion	Location	Macroscopic type	Size (mm)	Histology	pTNM	pStage	Operation time (min)	Estimated bleeding volume (mL)	Proximal margin (mm)	Post‐operative hospital stay (day)	Complications	Stenosis	Reflux
1	71	M	Remnant	Less	0‐IIa	10 × 20	Differentiated	T1a, N0, M0	IA	257	20	30	10	None	None	None
2	72	M	U	Less	0‐IIc	25 × 25	Undifferentiated	T1b2, N0, M0	IA	324	45	32	33	None	None	None
3	68	M	U	Less	Type 3	100 × 20	Undifferentiated	T3, pN3b, M0	IIIC	342	141	5	21	None	None	None
4	54	M	U	Less	Type 5	25 × 45	Differentiated	T3, N1, M1 (P1)	IV	354	114	5	10	None	None	None

Abbreviation: U, upper‐third.

**FIGURE 4:**
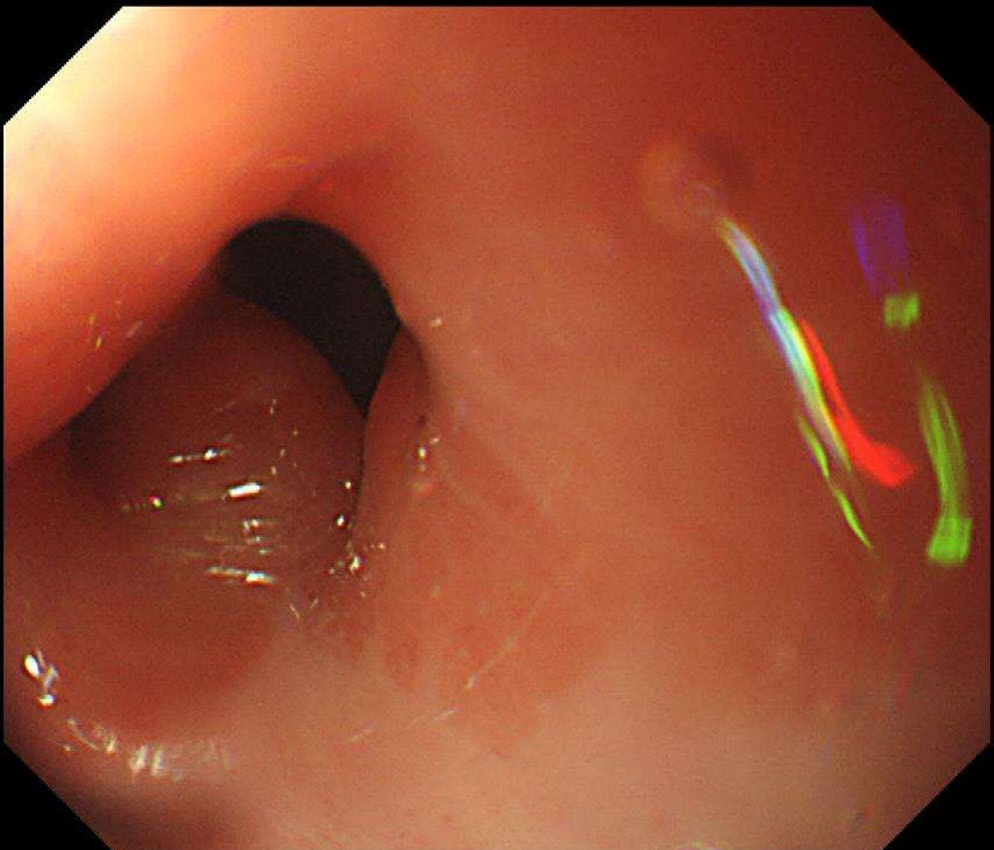
Endoscopic images of the esophagogastric junction closure site taken 1 year postoperatively.

Figure 4 presents endoscopic images of the esophagogastric junction closure site taken 1 year postoperatively. The endoscopic evaluation revealed no evidence of stricture or reflux esophagitis.

Regarding postoperative weight loss and nutritional markers, we excluded Case 4, a Stage IV patient who expired within 1 year after surgery. In the remaining three cases, the average weight loss rate at 6 months and 1 year postoperatively was 7.1% and 9.1%, respectively. Serum albumin levels at the preoperative, 6‐month, and 1‐year postoperative intervals were 3.8, 3.63, and 3.9 g/dL, respectively. The prognostic nutritional index (PNI) values at the same time points were 44.2, 42.7, and 45.8, respectively.

## 4 Discussion

The technique we report here enables gastrectomy with preservation of the fornix in patients with upper‐third gastric cancer located close to the esophagogastric junction, making securing a proximal margin distance with conventional subtotal resection difficult. Preserving the fornix and maintaining ghrelin secretion is expected to prevent postoperative weight loss and improve nutritional status [5].

Takagi et al. previously reported a surgical technique designed to avoid total gastrectomy [7]. However, their approach does not prioritize the preservation of the ghrelin‐secreting region and involves resecting the greater curvature. In contrast, SSTG aims to preserve the greater curvature as much as possible to maintain the ghrelin‐secreting region, resulting in a difference in the resection line due to these distinct concepts. A notable feature of SSTG is the partial resection of the right wall of the esophagus. Additionally, regarding reconstruction, SSTG involves making an incision in the right wall of the esophagogastric junction to resect the tumor. In contrast, the surgical technique reported by Takagi et al. extends the incision from the left wall of the esophagus to the gastric fundus, which is then used for anastomosis. These clear differences highlight that SSTG is a distinct surgical technique.

In terms of postoperative nutritional evaluation, Ojima et al. reported that the 6‐month postoperative weight loss rate was 8.7% following robotic subtotal gastrectomy and 15.8% after robotic total gastrectomy. In contrast, the postoperative weight loss rates regarding the present surgical method (SSTG) were 7.1% at 6 months and 9.1% at 1 year, demonstrating outcomes comparable to those after subtotal gastrectomy and superior to those following total gastrectomy. Moreover, serum albumin and PNI values recovered to levels equal to or exceeding their preoperative levels by 1 year postoperatively, indicating a favorable nutritional status.

Although the lower esophageal sphincter is partially destroyed, closure of the diaphragmatic crus prevents postoperative reflux, strictures, and other symptoms. The key point of this technique is to avoid completely severing the abdominal esophagus, ensuring that it remains connected at the angle of His. This finding is attributable to the fact that complete dissection can cause the esophagus to retract into the mediastinum, complicating suturing.

In addition, compared with total resection, areas near the tumor may necessitate further frozen section diagnosis. Preserving the left inferior phrenic artery is critical to maintaining adequate blood flow to the remaining portion of the stomach. Indocyanine green (ICG) fluorescence imaging is used intraoperatively to evaluate blood flow and confirm perfusion to the preserved stomach. This ensures that the residual stomach can be safely used for anastomosis, eliminating the need for esophago‐jejunal anastomosis, which is often challenging after total gastrectomy. Consequently, this approach is expected to reduce the incidence of postoperative complications. To date, this procedure, supported by ICG‐guided blood flow assessment, has been performed safely without any significant postoperative complications.

However, there are some possible concerns about the procedure. There might be a possibility of an inadequate surgical margin. To mitigate this risk, the resection margin was determined intraoperatively using endoscopy.

The traditional gastric cancer guidelines recommend a 5‐cm margin for advanced gastric cancer [8]. However, recent studies have reported that such a distance might not be necessary, as long as negative margins are confirmed through intraoperative frozen‐section analysis [9, 10].

During this procedure, the resection margin was determined intraoperatively using endoscopy. After resection, tumor‐free status was confirmed macroscopically. Additionally, the proximal margin was subjected to frozen section diagnosis to ensure negativity, achieving a balance between sufficient tumor removal and unnecessary resection. For instance, in patients No. 3 and 4, approximately 5 mm of the proximal margin was submitted for frozen section diagnosis. Consequently, the margin length reported in the permanent specimens, as shown in Table 1, appears shorter than the actual margin length. However, since negative margins were confirmed during surgery, the resection was deemed appropriate and adequately achieved. This method optimizes resection while ensuring oncological safety.

The second limitation could be cancer dissemination into the abdominal cavity. Since a portion of the bowel near the tumor site is opened during this procedure, meticulous attention is required to prevent leakage of intestinal contents. Complete suction by the surgical assistant is considered effective in preventing such leakage, thereby minimizing the risk of cancer dissemination into the abdominal cavity. To further reduce this risk, additional measures are planned for future surgeries. Specifically, the stomach will first be transected using an automatic stapler along the same resection line as the SSTG before opening the esophagogastric junction for anastomosis. Additionally, the resected side will be temporarily closed using intestinal clips before transection to prevent the spillage of gastric contents. These refinements aim to enhance oncological safety while maintaining the benefits of the procedure.

Third, the omission of lymph node dissection at stations number 2 and 4sa might be a limitation. Since the extent of resection and lymph node dissection was similar to that of subtotal gastrectomy, lymph node dissection at stations number 2 and 4sa was not performed. Song et al. [11] reported that metastasis to the number 2 lymph nodes is rare in early‐stage upper gastric cancer. This procedure targets gastric cancers localized to the lesser curvature without invading the anterior or posterior walls, where the likelihood of metastasis to the number 2 lymph nodes is low. Since this procedure is fundamentally aligned with subtotal gastrectomy, preserving the fornix is considered more critical compared with the prophylactic dissection of the number 2 lymph nodes. In each case, a preoperative assessment should evaluate whether the number 2 lymph node metastasis could be safely excluded. If intraoperative findings suggest the possibility of metastasis to the number 2 lymph nodes, conversion to total gastrectomy or proximal gastrectomy should be considered.

## 5 Conclusions

Our proposed SSTG technique enables the preservation of the fornix even in cases of upper‐third gastric cancer located closer to the esophagogastric junction than was previously possible.

## Author Contributions

**Kohei Fujita:** conceptualization, data curation, formal analysis, investigation, writing – original draft. **Hiroyuki Sagawa:** conceptualization, supervision. **Sunao Ito:** data curation, writing – review and editing. **Ryo Ogawa:** writing – review and editing. **Shuji Takiguchi:** supervision.

## Ethics Statement

The protocol of this research project has been approved by a suitably constituted Ethics Committee of the institution, and it conforms to the provisions of the Declaration of Helsinki.

Approval of the research protocol: The study was approved by the Institutional Review Board of the Graduate School of Medical Sciences, Nagoya City University. The approval number is 60‐18‐0008.

Registry and the registration no. of the study/trial: N/A.

Animal studies: N/A.

## Consent

Informed consent was obtained from the patient for publishing this case report and any accompanying images.

## Conflicts of Interest

Author Shuji Takiguchi is an editorial board member of the *Annals of Gastroenterological Surgery*.

## Supporting information


Video S1.

## Data Availability

All data generated or analyzed during this study are included in this article.
